# Patient-Provider Text Messaging and Video Calling Among Case-Managed Patients Living With HIV: Formative Acceptability and Feasibility Study

**DOI:** 10.2196/22513

**Published:** 2021-05-27

**Authors:** Virginia A Fonner, Samuel Kennedy, Rohan Desai, Christie Eichberg, Lisa Martin, Eric G Meissner

**Affiliations:** 1 Division of Global and Community Health Department of Psychiatry and Behavioral Sciences Medical University of South Carolina Charleston, SC United States; 2 Division of Infectious Diseases Medical University of South Carolina Charleston, SC United States; 3 College of Medicine Medical University of South Carolina Charleston, SC United States

**Keywords:** HIV, mHealth, text messaging, video calling, implementation science, mobile phone

## Abstract

**Background:**

Patient-provider communication is critical for engaging and retaining people living with HIV in care, especially among medically case-managed patients in need of service coordination and adherence support. Expanding patient-provider communication channels to include mobile health modalities, such as text messaging and video calling, has the potential to facilitate communication and ultimately improve clinical outcomes. However, the implementation of these communication modalities in clinical settings has not been well characterized.

**Objective:**

The purpose of this study is to understand patient and provider perspectives on the acceptability of and preferences for using text messaging and video calling as a means of communication; perceived factors relevant to adoption, appropriateness, and feasibility; and organizational perspectives on implementation within an HIV clinic in South Carolina.

**Methods:**

We conducted 26 semistructured in-depth interviews among patients receiving case management services (n=12) and clinic providers (n=14) using interview guides and content analysis informed by the Proctor taxonomy of implementation outcomes and the Consolidated Framework for Implementation Research. Participants were purposefully sampled to obtain maximum variation in terms of age and gender for patients and clinic roles for providers. The data were analyzed using quantitative and qualitative content analyses.

**Results:**

Most patients (11/12, 92%) and providers (12/14, 86%) agreed that they should have the capacity to text message and/or video call each other. Although consensus was not reached, most preferred using a secure messaging app rather than standard text messaging because of the enhanced security features. Perceived benefits to adoption included the added convenience of text messaging, and potential barriers included the cost and access of smartphone-based technology for patients. From an organizational perspective, some providers were concerned that offering text messaging could lead to unreasonable expectations of instant access and increased workload.

**Conclusions:**

Patients and providers perceived text messaging and video calling as acceptable, appropriate, and feasible and felt that these expanded modes of communication could help meet patients’ needs while being safe and not excessively burdensome. Although patients and providers mostly agreed on implementation barriers and facilitators, several differences emerged. Taking both perspectives into account when using implementation frameworks is critical for expanding mobile health–based communication, especially as implementation requires active participation from providers and patients.

## Introduction

### Background

Provider communication with patients outside of office visits can be critical to retaining people living with HIV in care, which is vital to sustaining health and preventing onward HIV transmission. In the United States, particularly in the southeastern United States where the HIV epidemic is now concentrated [1,2], rates of retention in care remain suboptimal. In South Carolina, approximately 20,000 individuals are living with HIV, but only 53% are retained in care [3].

Traditionally, providers, including case managers who have frequent interactions with patients regarding all aspects of clinical care, have relied on landline telephones to reach patients. These efforts can consume a considerable amount of provider time [4], especially for care involving chronic diseases [5] such as HIV. However, as technology has evolved, traditional means of communication have faced significant challenges, including people’s disinclination to answer a blocked or unfamiliar number and lack of a functioning voicemail box. Mobile technology has the potential to overcome these challenges and improve communication among patients experiencing barriers to care [6]. Enhancing patient-provider communication can work to build trust, foster more patient engagement, and improve health outcomes along the HIV care cascade [7-10].

Communication using mobile technology has become almost universal. Currently, 96% of American adults report owning a cell phone and 81% own a smartphone [11]. Mobile health (mHealth), defined as “medical and public health practice supported by mobile devices” [12], offers the potential to connect health care providers and patients, enhance in-clinic interactions, and improve care access. Studies have demonstrated that mHealth interventions implemented in HIV clinics are acceptable and can improve clinical outcomes [13-17]. However, many of these interventions either used automated one-way forms of communication (eg, appointment or medication reminders) or scripted two-way communication (eg, check-ins requiring confirmation of message receipt), and several studies have found no significant effect on clinical outcomes [18-20]. A recent systematic review found that more effective text messaging interventions allowed for interactivity, two-way communication, and links to support [21]. Several studies have also examined the feasibility and acceptability of video calling in the context of HIV, mostly to support telehealth visits [22], partner notification services [23], and counseling related to HIV testing [24]. There is a gap in understanding whether using mHealth modalities, including text messaging and video calling, simply as additional channels of communication between patients and providers in an unscripted, bidirectional manner could help improve communication without unduly increasing staff burden.

### Objectives

Implementing mHealth-based communication in a clinical setting presents unique challenges, as these modalities require adoption by both providers and patients. Although some HIV-related mHealth interventions have described participant and provider perspectives on acceptability and feasibility [25-27], few have used an implementation science approach [28]. Implementation science focuses on understanding how evidence-based interventions can be integrated into health-related policies and practices [29,30]. In this study, we sought to understand patient and provider acceptability of and preferences for expanding communication channels to include mHealth modalities within an HIV clinic in South Carolina using an implementation science perspective. The specific research questions were as follows:

1. Are text messaging and video calling acceptable forms of communication for patients and providers, and what are the preferred modalities?
2. What are the perceived benefits and barriers to adoption, appropriateness, and feasibility of expanding patient-provider communication to include text messaging and video calling?
3. From an organizational perspective, what factors will influence the implementation process?

## Methods

### Context

The research was conducted within an academic medical institution in Charleston, South Carolina, housing a Ryan White HIV clinic that delivers comprehensive outpatient medical care for approximately 1200 people living with HIV. A multidisciplinary team works within the clinic to provide clinical care, social support, and case management services. Approximately 20% of the clinic population receives case management consisting of the coordination of medical and social services tailored to meet individual clients’ needs. Patients are eligible for case management if they meet certain eligibility criteria established by the Ryan White Program (Part B), including having been diagnosed with HIV; income at or below 550% of the federal poverty level; and a demonstrated need for receiving assistance with social, community, legal, or financial barriers to care [31]. Case management has proven effective in improving clinical outcomes, including medication adherence and viral suppression [32].

This study was conducted as formative research to inform a subsequent intervention involving the provision of bidirectional text and video calling capabilities between case-managed patients and providers, specifically case managers and pharmacists, to enhance communication and ultimately improve retention in care and medication adherence.

### Participants, Sampling, and Recruitment

Patients were eligible to participate if they were aged 18 years or above, were receiving medical case management services through the HIV clinic, and reported having a cell phone capable of text messaging and/or video calling. Patients were recruited for the study immediately following their routine clinic visit and were purposefully sampled to obtain maximum variation in terms of age, gender, and race. Compensation was provided to the patients in the form of a US $50 gift card. Providers at the HIV clinic, including those providing medical services (eg, physicians, physician assistants, and pharmacists), social services (eg, case managers, social workers, and outreach coordinators), and administrative support (eg, nurse administrators, coordinators, and program support staff), were recruited via email. The providers were not compensated for their participation. The sample size was determined based on data saturation. The study was approved by the institutional review board of the Medical University of South Carolina. All participants provided verbal consent and gave permission to audiorecord the interviews.

### Potential Intervention Modalities Under Study

As part of the formative research development process, we sought to understand whether patients and providers would prefer using the standard SMS text messaging feature that is available on all cell phones or using an encrypted, secure app available only on smartphones and computers. The research team chose to use the secure messaging app, QliqSOFT (QliqSOFT, Inc), hereafter referred to as Qliq, as it provides secure text messaging for use within health care settings and offers a Health Insurance Portability and Accountability Act (HIPAA)–compliant platform that is free for patients [33]. The app is passcode protected, includes the ability to delete messages if a device is lost or stolen, and keeps contacts separate from contacts stored on the mobile device. For video calling, participants were also presented with a choice of a *standard* option, such as using *FaceTime*, or use of an encrypted app, although no specific encrypted video calling app was specified.

### Theoretical Orientation

We drew upon 2 implementation science frameworks to inform our study: (1) the Proctor taxonomy of implementation outcomes [34] and (2) the Consolidated Framework for Implementation Research (CFIR) [35]. We chose to focus on 4 of the 8 implementation outcomes outlined in the Proctor taxonomy, including acceptability (degree to which the intervention is agreeable, satisfactory, or has relative advantage), adoption (the initial decision, intent, or action to try an intervention), appropriateness (perceived intervention fit, relevance, or compatibility), and feasibility (extent to which an intervention can be conducted or successfully used) [34], as these were most salient to addressing our first 2 research questions and were most applicable to understanding factors relevant to implementation before intervention rollout. In addition, the Proctor taxonomy provides a flexible, practical framework for categorizing and understanding implementation aspects relevant to both providers and patients. We also used CFIR, a comprehensive, explanatory model of implementation addressing 5 major domains, including intervention characteristics, outer setting, inner setting, characteristics of individuals*,* and process [35]. CFIR is particularly relevant to understanding intervention implementation from an organizational perspective, which comprises our third research question.

### In-depth Interviews

Data collection consisted of semistructured in-depth interviews with both patients and providers. The interviews took place in a private room at the clinic and lasted for 30-60 minutes. Interview guides containing open-ended questions were developed with a focus on understanding preferences for, and acceptability of, a text-based intervention within the clinic (Multimedia Appendix 1). During the interviews, participants watched a brief demonstration video of Qliq and were asked about their initial thoughts and preferences of using Qliq versus SMS text messaging. Interview guides for providers were also developed using domains from the CFIR as a guide. In addition, at the end of the qualitative interviews, patients and providers were asked to verbally provide their responses to 4 statements using a 5-point Likert scale ranging from *strongly disagree* to *strongly agree*. The 4 statements were as follows: (1) “I would like to use this form of communication frequently,” (2) “Providers and patients should have the capacity to text and/or video call with each other,” (3) “Trying to implement a system to allow texting and/or video calling between patients and providers is too complex and not worth the time and risk,” and (4) “Most patients would be interested in communicating by text and/or video with their case manager or pharmacist.” All interviews were audiorecorded and transcribed verbatim by a third party.

### Analysis

Data were analyzed using both qualitative and quantitative content analyses [36,37]. Data on preferences for intervention modality (standard SMS text messaging vs encrypted secure messaging) were extracted from interview transcripts and quantitatively tabulated. Similarly, the Likert-scale statements relating to acceptability asked at the end of the in-depth interviews were analyzed quantitatively, with scores between patients and providers averaged by question and compared using two-tailed *t* tests. Quantitative analysis was performed using Stata version 16 [38].

For the qualitative analysis, emerging themes and technology preferences were discussed by the study team during data collection. Following the completion of data collection, we followed the steps outlined in the qualitative content analysis, including data preparation, categorization, and reporting [36]. All interview transcripts were read and discussed by 2 members of the study team. For data categorization, a coding scheme was developed using both inductive and deductive methods. Open, inductive coding was applied to the data regarding acceptability, barriers and facilitators to adoption, appropriateness, and feasibility. These codes were then further categorized and mapped onto the Proctor implementation framework. A second layer of deductive codes was applied to the data generated from provider interviews using predefined categories and constructs from CFIR. Data were coded using Atlas.ti (version 8). Memos were drafted to keep track of the categorization decisions, and the findings were iteratively discussed with the study team.

The research team consisted of a behavioral scientist trained in qualitative research methods (VAF), 2 research assistants (SK and CE) and a medical student (RD) with no affiliation to the HIV clinic and who received training in qualitative methods specific to the project, a research nurse who served as the study coordinator (LM), and a physician scientist who provided clinical care at the Medical University of South Carolina HIV clinic (EGM). Data collection and analysis were conducted by the study team members with no direct affiliation to the HIV clinic (VAF, SK, CE, and RD). Coding was conducted by a member of the research team (VAF), and a second member of the research team (RD) conducted an audit trail, which included reviewing transcripts, coding schemes, and memos to ensure conformability and authenticity. To enhance the trustworthiness and transparency of the data, authentic citations drawn from interview transcripts were used to create tables to report the results (Multimedia Appendices 2 and 3).

## Results

### Sample Characteristics

We conducted 26 interviews with 12 patients and 14 providers (Table 1). The patients were predominantly African American (10/12, 83%), aged between 23 and 57 years, and all reported having access to a smartphone. Providers included medical providers (6/14, 43%), social support providers (5/14, 36%), and support staff (3/14, 21%).

**Table 1:** Sample characteristics.

Characteristic				Value
**Patients (n=12)**				
	**Gender, n (%)**			
		Male	5 (42)	
		Female	6 (50)	
		Gender fluid	1 (8)	
	**Race, n (%)**			
		African American	10 (83)	
		Native American or White	1 (8)	
		White	1 (8)	
	Age (years), mean (SD)		39.5 (9.8)	
	**Smartphone access, n (%)**			
		Yes	12 (100)	
		No	0 (0)	
	Years enrolled in clinic, mean (SD)		6.4 (4.3)	
**Providers (n=14), n (%)**				
	**Role**			
		Medical provider	6 (43)	
		Social support provider	5 (36)	
		Support staff	3 (21)	
	**Gender**			
		Male	3 (21)	
		Female	11 (79)	

### Acceptability

Most patients (11/12, 92%) and providers (12/14, 86%) either strongly agreed or agreed with the statement that they “should have the capacity to text and/or video call” each other. Similarly, participants mostly agreed that patients would be interested in texting and/or video calling providers, specifically case manages and/or pharmacists, and felt that implementing texting messaging and/or video calling was “worth the time and risk” (Table 2). No statistical differences were found between patients’ and providers’ perceptions of acceptability.

**Table 2:** Acceptability of text messaging and video calling between patients and providers.

Statement	Participants, mean (SD)^a^	Providers, mean (SD)^a^	*P* value
“I would like to use this form of communication frequently”	4.4 (0.9)	4.2 (1.0)	.48
“Providers and patients should have the capacity to text and/or video call with each other”	4.4 (0.8)	4.0 (0.9)	.22
“Trying to implement a system to allow texting and/or video calling between patients and providers is too complex and not worth the time and risk”	1.7 (0.8)	1.9 (0.5)	.30
“Most patients would be interested in communicating by text and/or video with their case manager or pharmacist”	4.3 (0.6)	4.5 (0.6)	.52

^a^Likert scale ranged from 1 (strongly disagree) to 5 (strongly agree).

In general, patients felt that communication with their health care team was already quite good and that being able to text or video call would further enhance their ability to connect. When describing the potential benefits of being able to text with a case manager, one participant said:

Texting is fast, go...it gets straight good, easy for you to reply back. Any questions, just [text]...and then it probably might open up the dialogue a little bit more.

Providers, especially social support providers, expressed frustration with their ability to reach patients using landline telephones. One social service provider said:

There are so many patients that I have...where I am not able to get ahold of because they don’t have voicemail set up. Their voicemail is full. They might have called me, but when I try to call them back, I can’t get them for whatever reason. So that’s very frustrating. It’s very time consuming. I want to be an efficient [position redacted], and when doing a lot of phone tag takes up a bulk of your day when you have other patients waiting on you to get to them, it’s just a nuisance.

Providers mostly felt that having the ability to text patients would help alleviate this challenge.

### Preferences

With regard to preferring SMS text messaging to Qliq, the secure messaging app, 83% (10/12) of patients endorsed a preference for Qliq (Table 3). For providers, preferences were more varied. Qliq was favored over SMS text messaging (5/14, 37% vs 3/14, 21%, respectively), although a sizable proportion either had no preference (2/14, 14%) or a preference for having both SMS text messaging and Qliq available (2/14, 14%) to reach as many patients as possible. In other words, providers, specifically most social service providers, were deferential toward patients’ preferences. One social support provider voiced:

I just think that I’m kind of torn between both [using Qliq or SMS]...I think it’s more about privacy for the client, but it’s also convenient for us.

In addition, 14% (2/14) of providers felt that adding a text messaging platform as a form of communication, including either SMS text messaging or Qliq, was duplicative of the secure messaging function built into the existing patient portal to which patients already had access, called MyChart. Those favoring the patient portal were primarily medical providers who frequently interacted with MyChart as part of their clinical duties. Of those favoring Qliq, both patients and providers highlighted the security features and enhanced privacy, and patients liked other features of Qliq, such as the ability to see when a message had been read. Among those favoring SMS text messaging, convenience was cited as the primary reason. Patients’ and providers’ preferences for SMS text messaging versus Qliq mirrored their preference for video calls, that is, those favoring SMS text messaging typically favored using FaceTime, and those favoring Qliq typically preferred a similar secure app for video calls. Some patients and most medical providers did not see a need for video calling and therefore did not have a preference for either method.

**Table 3:** Patients’ and providers’ preferences for SMS text messaging versus Qliq.

Provider preference for text messaging modality	Patients’ preferences (n=12), n (%)	Providers’ preferences (n=14), n (%)
Prefers SMS text messaging	2 (17)	3 (21)
Prefers secure app (Qliq)	10 (83)	5 (37)
Prefers use of the web-based patient portal (MyChart)	0 (0)	2 (14)
Prefers that both methods be available (SMS text messaging and Qliq)	0 (0)	2 (14)
No preference	0 (0)	2 (14)

### Perceived Benefits and Barriers to Adoption

Patients and providers agreed on the perceived benefits of text messaging, which included efficiency, convenience, and ease of use, irrespective of modality (Qliq vs SMS text messaging; Multimedia Appendix 2). The ability to engage in asynchronous conversations through text messaging was seen as a clear advantage by both groups. Patients specifically cited the inability to answer calls while at work and hesitation to answer calls because of privacy concerns related to HIV. One participant stated:

As a patient, we go through our day with people and doing things. And people don’t really know about our situations. So a lot of people feel, when they do get a pop-up phone call, they can’t really answer the phone and speak freely because of who they’re around.

Although providers also felt that having the ability to text would be efficient and convenient, some worried that these benefits could become drawbacks by creating unreasonable expectations of instant access and overutilization by patients, thus leading to increased workload. A few other barriers were mentioned. Some patients and providers cited the impersonality of texting as a barrier. As a medical provider voiced, “people don’t see my concern over text.” For this reason, a few participants preferred video calling over text messaging. One participant said:

Video chat I think would be better, like I said, that way you can visually see them and try to get body language and stuff like that, communicating, that’s means a lot.

Some providers and patients also mentioned cost and cell phone access, specifically smartphone access, as potential barriers.

### Appropriateness

From both patients’ and providers’ perspectives, allowing text messaging and video calling was perceived as appropriate because of its potential ability to improve communication while being relatively safe and not overly burdensome (Multimedia Appendix 2). Most patients articulated a desire for texting, and many providers were enthusiastic about reaching patients using this modality, as it was perceived to be mutually beneficial. When describing her preferences for text messaging (Qliq vs SMS), one social support provider said:

Whatever will make my work life easier. Again, when it comes to the client, it’s about making sure that they get what they need.

The appropriateness of video calling was less clear for both the patients and providers. However, for providers who field questions about medication and adherence, such as pharmacists, video calling was perceived as offering the distinct benefit of being able to see which pill a patient had questions about and to directly observe a patient taking their medication in cases where daily adherence was in question. Some social service providers also felt that video calling would be useful for providing social support and counseling, as it would enable visual contact.

### Feasibility

Two issues regarding feasibility emerged: (1) privacy or security issues and (2) concern over message content (Multimedia Appendix 2). With regard to privacy and security, patients and providers were well aware of the stigmatizing nature of HIV and were in agreement that nothing directly mentioning HIV should be sent over text. Providers were concerned about patients sending protected health information over text, whereas patients did not share this concern. One social support provider explained:

...You can control what you send out to patients, but you can’t control what patients send back to you.

Patients mostly trusted their health care providers to be discreet, although there was some concern about how their contact details would be stored in the providers’ phones and whether the providers’ phones would be used outside of the office setting. Some patients also had privacy concerns over video calling because of the potential of being overheard when discussing sensitive health-related matters. For text messaging, patients favored Qliq, the secure app, in large part because of the enhanced security features. One patient stated:

You have kids, you have family members who doodle in your phone. They can read the text, everybody else’s text. But when it comes to your health and your privacy, there should be an app that is created just for that.

With regard to message content, providers agreed that texting should be used for short, simple messages, such as checking-in with patients or confirming logistics for transportation to a clinic visit. Patients mostly agreed to use text messaging for such short messages, although some thought it would be helpful if clinical information, such as laboratory results, could also be sent over text.

### CFIR-Specific Implementation Constructs

Provider-specific themes relevant to CFIR mainly fell into 3 domains: (1) intervention characteristics; (2) the inner setting, specifically the implementation climate; and (3) the outer setting, specifically patient needs and resources (Multimedia Appendix 3). With regard to intervention characteristics, providers, specifically social support providers, mostly felt that texting offered a significant relative advantage to the status quo in that it would both reduce the *phone-tag* burden and would increase efficiency. However, some worried that having the ability to text and video call would be complex to implement, as it could lead to significant disruptions and task them with yet another mode of communication to manage throughout the day. Providers felt that the source of the intervention was internal and had been developed to respond to patient needs and provider frustrations while trying to communicate with patients using traditional modalities.

With regard to the inner setting, there was a discernable tension for change raised by a number of providers, specifically those associated with providing case management and outreach services. These providers already had access to basic cell phones (*flip phones*) for patient communication but found texting on these devices cumbersome; hence, providers felt that smartphone access was needed to facilitate text-based communication. Importantly, one provider felt that using a secure app for text messaging with patients would be *overkill* and unnecessarily complicate the process of text messaging. However, most providers felt that the enhanced security measures of Qliq were of value, both to themselves and patients, and offered benefits over standard SMS text messaging.

With regard to the outer setting, providers, especially social support providers, were keenly aware of patient needs and perceived text-based communication as a means to better meet patient needs. Providers’ perceptions of patients’ needs were mostly accurate, although a few differences emerged. Several providers felt that patients would prioritize convenience over security and were worried about those without smartphone access. One social support provider said:

I think also knowing the majority of our patients and their tech savvy skills, I think texting [SMS] would be much easier for them...not all of them [patients] are gonna have smartphones, unfortunately.

In addition, several providers felt that patients already had the ability to send secure text messages to providers through the web-based patient portal and thus viewed expanding text messaging capabilities as redundant. However, patients consistently cited logistic difficulties using the patient portal (eg, trouble remembering passwords). One patient voiced:

I see [text messaging] as a better improvement ‘cause when I go through the patient care app like every six months you gotta get a new password and it’s like, “I don’t want to get a new password. Just show me when the next doctor’s appointment is coming up.”

Some providers were aware of these difficulties and were concerned that using a secure app for texting would result in similar logistical challenges.

## Discussion

### Principal Findings

In this formative study of 26 patients living with HIV and HIV care providers within a South Carolina–based clinic, we found high acceptability and feasibility for expanding patient-provider communication channels to include text messaging and video calling. Below, we discuss the implications of our findings for each specific research question.

### Acceptability and Preferences for mHealth Communication Modalities

The high acceptability of text messaging found in our study resonates with the findings from prior mHealth interventions implemented in HIV clinics that used either SMS or a form of secure messaging [20,26,27,39]. To our knowledge, our study is one of the first to directly compare preferences for standard versus secure text messaging among patients living with HIV and providers. Although we did not find unanimous consensus, patients’ and providers’ overall preference for using secure messaging because of privacy concerns mirrors prior insights regarding technology use, stigma, and privacy among people living with HIV [40]. However, enhanced security comes at the cost of additional requirements (eg, smartphone use) and logistical hurdles (eg, passwords), which have been identified as barriers to technology use [41,42]. Therefore, there is a need to consider issues surrounding security, access, and convenience when developing best practices related to the use of text messaging within HIV clinics. Recently, efforts have been made to clarify best practices regarding text messaging use in health care settings [43-45]. For the intervention informed by this formative study, the clinic ultimately chose to use the secure messaging app but also allowed for standard SMS text messaging for patients without smartphones to maximize both security and access.

### Benefits and Barriers to Adoption, Appropriateness, and Feasibility

We found that patients and providers agreed that text messaging could be a faster, more efficient mode of communication and seemed appropriate given its potential to increase patient engagement. There was less consensus on the benefits of video calling, although both patients and providers saw its utility in specific circumstances, such as allowing for directly observed therapy and fostering social support in times of need. Our findings are supported by other studies that have examined patients’ and providers’ perspectives on mHealth among people living with HIV, specifically with regard to the perceived benefits of convenience and ease of use and the perceived barriers of cost or access and confidentiality or security concerns [25,28].

What is novel from our findings is that providers’ acceptance of and enthusiasm for using the technology was often contingent upon patients’ ability and willingness to use it. With regard to technology preference, many providers were deferential to patients’ preferred modality (SMS text messaging vs Qliq). In addition, patients factored in providers’ use of the technology (eg, storage of contact details in provider phone and location of phone use) into their assessment of feasibility.

Given our study’s findings of patient-provider interdependence related to intervention acceptability, more theoretical understanding is needed concerning the implementation of mHealth interventions dependent upon patient-provider interaction. Although many implementation science frameworks include a construct related to patient needs, few, if any, elevate the importance of this construct to the forefront. One recent study to understand the implementation of patient-centered care transformation interventions made adjustments to CFIR throughout their analysis, including promoting *patient needs as resources* to its own domain as opposed to a subdomain of the *outer setting*, which investigators felt reflected the prioritization of patients and patient satisfaction currently being emphasized within health-related interventions [46]. Although this change is notable, gaps remain in understanding health interventions whose effectiveness depends on uptake and use among both patients and providers and on the interplay between them.

### Organizational Factors Relevant for Implementation

Providers perceived that adding text messaging and video calling as communication channels was an internally generated idea to overcome frustrations with the existing channels (eg, using landline phones). Some staff were concerned that the very features that made text messaging appealing, such as its convenience and ease of use, could create false expectations of instant access and overutilization, thus creating implementation challenges by disrupting existing workflows and increasing staff burden. However, most felt that the benefits of improved patient engagement and communication outweighed the potential drawbacks.

Results from our formative study show that patients’ and providers’ intentions to use text messaging and video calling exist on a continuum, highlighting that one size may not fit all. For example, although many providers were enthusiastic, several providers expressed reticence to use text messaging and/or video calling with patients, mostly as a result of failing to see the advantage over existing platforms or because of concerns that the intervention would increase workload and reduce efficiency. However, if interventions such as offering text messaging and/or video calling were to be rolled out clinic wide, uneven use of the intervention might result in technology preference mismatches, for example, patients who prefer texting assigned to providers who prefer to call and vice versa. This finding suggests that implementation strategies are needed to ensure that patients and providers are aware of and can access all possible communication methods, paying specific attention to patient preferences.

### Limitations

Our study has several limitations. The generalizability of our findings might be limited, given that the data came from one specific HIV clinic in South Carolina. However, provider and patient participants were purposefully chosen to maximize variation in terms of professional position and sociodemographic characteristics, respectively, to capture a diverse set of experiences and perceptions. Although not formally tracked, almost all patients and providers who were invited to join the study agreed to participate. Our sample predominantly consisted of African American patients, which reflects the racial makeup of the clinic population more generally and the disproportionate burden of HIV found among this group. Contributing to this limitation, our sample size was relatively small, and we were unable to tease out the differences in patient perspectives by specific characteristics, such as age, race, and place of residence (urban vs rural), despite known disparities in technology use across such factors [47-49]. This limitation also highlights the need to recognize that preferences for communication differ among individuals, and allowing choice is important.

In addition, patient participants were selected from case-managed patients who were physically present at the clinic to attend their regular medical appointments. Although all case-managed patients demonstrated a need for supportive services to help them stay engaged in care, we did not specifically sample patients according to their medication adherence status or past clinic attendance. We are also missing perspectives from patients who are disengaged from medical care—a critical group to reach. However, we did receive provider perspectives from outreach staff who specifically work to reengage patients in care and who spoke on the potential benefits and challenges of using text messaging and video calling to communicate among this population. Owing to logistical constraints, we were unable to return transcripts for member checking, although the findings were informally discussed with the providers during intervention development. In addition, all patients in our sample had access to smartphones, whereas smartphone access is not universal among the clinical population. Finally, our study took place before the implementation of the text messaging and video calling intervention; therefore, the findings are based on perceptions of hypothetical use and barriers to implementation. Implementation findings gathered after the completion of the intervention will help convey a more complete understanding.

### Conclusions

We found broad enthusiasm, acceptability, and feasibility for the implementation of bidirectional text messaging between patients and providers, with a preference for doing so using secure means. There was less consensus about the appropriateness of video calling, although both groups acknowledged its utility in certain circumstances. Engagement in text messaging and video calling within an HIV clinic setting requires buy-in from patients and providers. The expansion of communication channels will only be effective in enhancing communication if the services are used by both parties. Implementing a bidirectional intervention raises important questions for implementation research, as it requires a thorough understanding of the organizational, provider, and patient needs and finding implementation strategies and options that work for all.

## Appendix

Multimedia Appendix 1 Semistructured in-depth interview guides.

Multimedia Appendix 2 Exemplary quotes related to implementation outcomes using Proctor's taxonomy.

Multimedia Appendix 3 Exemplary quotes related to Consolidated Framework for Implementation Research–specific domains and constructs.

## Abbreviations

CFIR: Consolidated Framework for Implementation Research
HIPAA: Health Insurance Portability and Accountability Act
mHealth: mobile health
