# Supplementary material for: Patient-Provider Text Messaging and Video Calling Among Case-Managed Patients Living With HIV: Formative Acceptability and Feasibility Study
Source: JMIR Form Res. 2021 May 27;5(5):e22513. doi: 10.2196/22513 (PMC8193483; doi:10.2196/22513)
Supplement: Multimedia Appendix 1 [file formative_v5i5e22513_app1.docx]

**Supplementary Appendix**

**A. In-depth interview guide: Clinic staff**

**Part 1**

1. Please briefly describe your role here at the clinic.
2. Please tell me about how the idea of the text/video intervention came about, if you are aware (probes: limitations of current ways of communication?; who developed it?; your role in development?)
3. To what extent do you think the text/video intervention would be helpful for case managed patients?
   1. For what reasons? (Probes: perceived patient needs?, ability of intervention to meet patients’ needs?; relative advantage over status quo or other possible solutions?)
   2. What are potential advantages of the intervention for you? For patients? For the clinic?
   3. What are potential disadvantages or risks of the intervention for you? For patients? For the clinic?
   4. How effective do you think this intervention will be in the clinic?
      1. For what reasons? (probes: work culture?, clinic environment?, patients’ use of it?, impact on workflow?)
   5. How receptive have staff been to the proposed intervention in the clinic?
   6. What privacy concerns or risks do you have about texting with patients? (probes: privacy concerns for you as staff?, perceived privacy concerns of patients?).
   7. What are potential barriers to implementation? (probes: workflow, additional workload) Potential facilitators? (probes: how to get staff and patients on board)
4. What sorts of information do you think would be appropriate for patients and their case managers to communicate over text or video? What sorts of information do you think would be inappropriate?
5. How would you describe the work culture here in the clinic?
   1. In the past, how easy or difficult has it been to implement change. For what reason(s)?
   2. How do you think the culture here could affect implementation of this intervention?
   3. Do you know of any other organizations/clinics that are implementing something similar? If so, please describe.
6. How well does the intervention fit with existing work processes and practices in your setting?
7. When considering approaches to communicating by text and/or video with patients, which issues do you think are more important?
   1. For example, using standard test messaging platforms may be user friendly and easy, but may be less secure. Or the other hand, using a dedicated, secure application may have more layers of protection, but also may take more time for patients to utilize (i.e. password protected). What do you think most patients would feel is more important? For what reasons?
   2. For regular texting you would need to communicate by having a work cell phone. Many of the apps offer desktop versions where you could communicate with patients using your computer (no cell phone required). Which method (regular texts vs. apps) do you prefer? For what reasons?
8. Do you think patients would have any difficulty avoiding use of personal health information (for example names) over unencrypted text messaging platforms?
9. How ideally would patient identity be stored on your phone to facilitate your ability to keep contacts straight while minimizing the risk of inadvertent disclosure of patient information? Would you prefer patients’ names be used? Or do you think it is better to find a confidential way to store patient contact information on cellular devices? What suggestions do you have?

**Part 2**

***Now I am going to give you a brief demonstration of a specialized application that would allow you to communicate with your patients by text. I will ask you your thoughts about it afterwards, and ask you whether you think using an application like this or standard text messaging platforms would be your preference for communicating with patients. [Conduct demonstration of free demo version of qliqsoft application***, *https://www.qliqsoft.com/]*

1. What are your initial reactions to the application?
   1. What do you like about it? (probes: design, usability, interface, etc.)
   2. What do you not like about it? (probes: design, usability, interface, etc.)
   3. How complicated is the app to use for you? For what reasons?
   4. How complicated is the app to use for patients? For what reasons?
2. What supports, such as online resources, standard operating procedures, do you think need to be developed to help you implement and use the app?
3. What concerns, if any, do you have about having this app on your phone/computer?
4. How likely would you be to use this application? (probes: how often would you communicate, how often would you want patients to communicate with you? What types of communication?)
5. How do you think this app will change how you perform your job here at the clinic?
6. Would you prefer using this app to communicate with patients, or plain text messaging. For what reasons?
7. What about communicating with patients through video. Would you prefer using FaceTime, or an encrypted, HIPAA-compliant app, similar to qliq?

***We are almost done. Before we end the interview, I would like to ask you several brief questions about the capacity to text and video with patients, if this approach was made accessible to you. Please respond to each statement with one of the following: Strongly disagree, disagree, neither disagree or agree (neutral), agree, or strongly agree. Feel free to elaborate on your answer.***

1. I think that I would like to use this form of communication frequently.
2. I think providers and patients should have the capacity to text and video with each other.
3. I think trying to implement a system to allow texting and video communication between patients is too complex and not worth the time and risk.
4. I think most patients would be interested in communicating by text or video with their case manager or pharmacist.

Finally, are there any other thoughts about this subject that you’d like to share with us, but which we have not asked you about during this interview?

***Thank you for taking the time to speak with me today. We are going to use your feedback to improve and guide the implementation process.***

**B. In-depth interview guide: Patients**

**Part 1**

1. As a patient, what are the benefits of receiving case management services at this clinic?
2. What would or could improve case management services at this clinic?
3. What challenges do patients such as yourself face regarding access to care at this clinic?
4. What types of issues are the case management team most helpful in addressing? Least helpful?
5. Please tell me about how you currently communicate with your case manager? Social worker? Pharmacist? Other members of the case management team?
   1. What challenges have you had?
   2. What do you like about how you communicate with your case management team?
   3. What would help make communication with your case management team better?
6. Are there any challenges to how the clinic staff are currently able to communicate with you or how you are able to communicate with them? What are they?
7. How would you feel about communicating with your case management team by text? By video?
8. Would you prefer using a specific application (“app”) for text messaging with your case manager that would help keep all communication secure, or plain text messaging (like what you already use to text with family and friends)? For what reasons?
9. Would you have any worry about accidentally disclosing your HIV status if you started text messaging or video conferencing with your case manager or the clinic pharmacist? Please explain.
10. What would you think are some potential challenges to using text/video to interact with your case management team?
11. What would help or encourage you to use text/video to interact with your case management team?
12. What types of messages would you want to receive from your case manager? What types of messages would you want to send? What types of information, if any, would you want never mentioned in a text with your case management team?
13. What concerns do you have (if any) about communicating with your case management team by text? By video? (probes: privacy, potential for others to see text messages?)
14. How would you want your identity stored on your case managers phone? Would you prefer they use your name? Or would you prefer they find a confidential way to store your contact information on their cellular device?
15. What other suggestions do you have for improving how you communicate with your case management team, or care at this clinic?
16. Now I would like to ask you about your own technology use.
    1. What type of phone do you have?
    2. How often do you use it? And how do you use it (e.g., calls, texts, internet, games)?
    3. Are there times in the past when you have not been able to use your phone (e.g., ran out of minutes, stolen, borrowed by a friend)? Please describe.
    4. How do you currently use your phone to communicate with other healthcare providers, like your primary care physician, dentist, etc.?

**Part 2**

***Now I am going to give you a brief demonstration of a specialized application that would allow you to communicate with your case management team by text. I will ask you your thoughts about it afterwards, and ask you whether you think using an application like this or standard text messaging platforms would be your preference for communicating with your care team. [Conduct demonstration of free demo version of qliqsoft application***, *https://www.qliqsoft.com/]*

1. What are your initial reactions to the application?
   1. What do you like about it? (probes: how easy to use, look of it, privacy)
   2. What do you not like about it? (probes: how easy to use, look of it, privacy)
   3. How complicated is the app to use? (probes: do you think you could use this app to communicate with your case manager without much difficulty?)
2. What concerns, if any, do you have about having this app on your phone?
3. How likely would you be to use this application? For what reasons?
   1. How often do you envision using this app to communicate with your case management team? (probes: use to communicate about appointments, problems, etc.)
   2. How often would you want your case management team to reach out to you using this app? (probes: before appoints, if appointments are missed, other times?)
   3. What types of communication would you use this app for? (probes: what would you want your case manager writing you about, and what would you write to your case manager about?)
4. How do you think this app would change the care you receive at this clinic?
5. How could staff here help encourage patients to use this app?
6. Would you prefer using this app, or plain text messaging? For what reasons?
7. What about communicating with your case management team through video. Would you prefer using FaceTime, or an encrypted, HIPAA-compliant app, similar to qliq?
8. What questions do you have for me?

***We are almost done. Before we end the interview, I would like to ask you several brief questions about how you feel about using text and video with your case managers and other clinic staff, if this approach was made accessible to you. Please respond to each statement with one of the following: Strongly disagree, disagree, neither disagree or agree (neutral), agree, or strongly agree. Feel free to elaborate on your answer.***

1. I think that I would like to use this form of communication frequently.
2. I think providers and patients should be able to text and video with each other.
3. I think trying to change the system to allow texting and video communication between patients is too hard and not worth the time and risk.
4. I think most patients would be interested in communicating by text or video with their case manager or pharmacist.

***Finally, may I ask your age, ethnicity, and length of time you have been at this clinic?***

Age: _______

Race and Ethnicity: _________

Length of time in care at clinic: ________

Gender: ________

***Thank you for taking the time to speak with me today. We are going to use your feedback to improve and guide the implementation of this process of communication.***
