# Supplementary material for: Patient-Provider Text Messaging and Video Calling Among Case-Managed Patients Living With HIV: Formative Acceptability and Feasibility Study
Source: JMIR Form Res. 2021 May 27;5(5):e22513. doi: 10.2196/22513 (PMC8193483; doi:10.2196/22513)
Supplement: Multimedia Appendix 2 [file formative_v5i5e22513_app2.docx]

Appendix 2. Exemplary quotes related to implementation outcomes using Proctor’s taxonomy

| **Aspect of implementation** | **Sub-category** | **Exemplary quotes from patients** | **Exemplary quotes from providers** |
| --- | --- | --- | --- |
| **Perceived benefits of adoption** | Quick/efficient | “I would be very likely to use the app…that way you're not stopping what you're having to do, it's just quick, message.” *–White gender-fluid patient, 38 years old* | “It think it will speed up the time that it takes for us to actually connect with the client …Sending them a text is quicker, and while it's on your mind you type whatever you need. Send it, shoot it, it’s quicker communication.” *–Social support provider* |
|  | Convenience | “Well that’d [text messaging] be good, especially on like days when I’m at work. Instead of that phone ringing and I’m like ‘Oh, I can’t.’ This way here [acts like checking a text], ‘Oh okay.’” *–African American female patient, 57 years old* | “It's [text messaging] an easier way to reach our clients.” *–Social support provider* |
|  | Ease of use | “It's pretty simple…It’s easy.” *–African American female patient, 47 years old* | It [Qliq] seems pretty easy...I really like it's pretty similar to how you would normally text. I mean, it looks pretty user-friendly.” *–Medical provider* |
| **Perceived barriers to adoption** | Impersonality of text messaging | “I like it when we're able to talk in person. That's really helpful because I can see expressions.” *–White gender fluid patient, 38 years old* | “I feel as though texting loses emotion…People don’t see my concern over text.” *–Medical provider* |
|  | Expectations of instant access (provider only) | N/A | “…Texting implies that you have my full attention and I’m at your disposal anytime, anywhere.” *–Medical provider* |
|  | Potential for overutilization (provider only) | N/A | “it would be my hope that the patients would be informed that while this [text messaging] is a mode of communication, it's not meant to be over-utilized. Like, please don't send me 15 text messages in one day.” *–Social support provider* |
|  | Cost and access | “…some people have monthly bills for phone plans. They have unlimited. Some people have minutes and stuff.” *–African American male patient, 33 years old* | “…Sometimes when we do contact them [clients] via phone, their phone's turned off a lot. They haven't made their bill…That's one of the disadvantages I think, is the financial thing that a patient can't afford the technology.” *–Social support provider* |
| **Appropriateness** | Desire for texting (patient)  Meeting patients where they are (provider) | “…phone calls get drawn on and then get still and I be like ‘okay miss, is that it?’…I know she [case manager] only calling for my best interest, but I am not the phone talking guy, person. I would rather text all day.” *–African American male patient, 26 years old* | “If that’s the way you [the patient] wants to communicate with me, I’m all for it. If it will get you to answer my questions, you do what I what you to, then sure.” *–Social support provider* |
| **Feasibility** | What information to send/not send by text | “I would think just a short check in as far as text …like, "Hey, are you doing okay? Is there anything I can help with?*"   – White gender-fluid patient, 38 years*  …She'll [the case manager] get information about my lab work before I do…she could just send me a text message and be like ‘hey this changed, this improved’” –African American male, 26 yrs | “Just a simple text and not a detailed text. We're not trying to give a dissertation about the client’s medical history. We just want to give them simple instructions.” *–Social Services Provider* |
|  | Privacy/security | “You have kids, you have family members who doodle in your phone. They can read the text, everybody else’s text. But when it comes to your health and your privacy, there should be an app that is created just for that.” *–African American male patient, 37 years old* | “…You can control what you send out to patients, but you can't control what patients send back to you.” *—Social support provider* |
