# Supplementary material for: Patient-Provider Text Messaging and Video Calling Among Case-Managed Patients Living With HIV: Formative Acceptability and Feasibility Study
Source: JMIR Form Res. 2021 May 27;5(5):e22513. doi: 10.2196/22513 (PMC8193483; doi:10.2196/22513)
Supplement: Multimedia Appendix 3 [file formative_v5i5e22513_app3.docx]

Appendix 3. Exemplary quotes related to CFIR specific domains and constructs

| **CFIR domain** | **Construct** | **Summary** | **Exemplary quotes** |
| --- | --- | --- | --- |
| **Intervention Characteristics** | Complexity | Some providers were concerned that having to text with clients would increase their work load, or make work harder. However, the majority of staff felt that text messaging would be easy to implement, reduce their workload and be more efficient (see potential benefits to adoption) | “…this [app] wouldn't be hard to use for staff, pretty straightforward to me. I think that it's simple. It's secure, and I don't think they [staff] would have a problem learning it.” *--Social support provider*  “[Workflow issues] tends to be anything that takes me longer, I'm not efficient at…That's a threat that anything like this [texting with patients] can ... Well, now I have to answer texts from my patients, Monday through Thursday, and Friday night, and any old time on the weekend. Is that going to slow me down?” *–Medical provider* |
|  | Relative advantage | Most providers felt that having the ability to text with patients would be better than the status quo as it would make connecting with patients easier and would provide better access. A minority felt offering texting was duplicative of existing services, such as the patient portal, or offered no distinct advantage. | “I think for me, I still think that it [text messaging] would give us opportunity to engage our clients more, to get them in, to care more, good teamwork…I think it gives us more productivity and better opportunity for safety and all that. I just think that it's a good thing.” *–Social support provider*  “I think that they [patients] have better access to me [through text messaging]. You wouldn't have to play phone tag.” *–Medical provider*  “...is it duplicating what's already available? And also apps take up storage and why wouldn't they [patients] just delete it if they want something else.” *–Social service provider* |
|  | Intervention source | Providers felt the intervention was developed internally and arose from existing challenges with communicating with patients | “…People [staff members] have talked about how good it would be to be able to, first of all, to have iPhones but then with the ability to text easily but also perhaps to be able to communicate with particularly younger people in a way that is more comfortable for them and a way that they're more likely to use and utilize.” *–Social support provider* |
| **Outer Setting** | Patient needs and resources | Meeting patient needs was the driving factor behind the intervention development. Providers were aware of patients’ desire for texting and concern for privacy. A few differences emerged, such as that some providers felt patients would prioritize convenience over security and some felt patients already had the ability to text with providers through the patient portal. | “You know that people will text, but maybe they’re not answering their phone, maybe they don’t want to talk…they would just rather text…” *–Social support provider*  “…I think patients want to feel privacy. I think that's number one because I think especially if you have someone that is, I think having something that's more secured would be good because I think they feel safer that way.” *–Social support provider*  “I think patients would just want to text like normal, but for us, I don't think we'd be able to do that. I think we'd have to have a special app that was HIPAA protected, encrypted, or whatever, in order to protect us and the patients.” *–Medical provider* |
| **Inner setting** | Compatibility (Intervention climate) | Most providers, especially social support providers, felt that text messaging fit well within the organizational culture and the overall mission to communicate with patients and meet their needs. Other providers felt they already communicated with patients using various modalities that catered to patients and adding more would overburden existing work flows. | “It's easy for me, I'm at work. It's easy for me to receive a text and text back, versus me answering the call. Then I forget to call you back. It [the text messaging intervention] came up where maybe we need to do something in regards to that.” *–Social support provider*  “…we get calls from the pharmacy, the patients. We're also making calls to them every day or emailing them…and I just can't imagine having to deal with that [text messaging] in addition to all the traffic we get.”  *–Social support provider* |
|  | Tension for change (intervention climate) | Among providers offering social support services, there was as an understanding that the current situation was untenable, and that new interventions, specifically the provision of smartphones, was needed to improve communication with case-managed patients. | “…because what the outreach group is using [to communicate] is not satisfactory. They have flip phones, which are impossible to use, and they need to have better access to be able to communicate with patients who are out of care.” *–Support staff*  “…It's frustration…Patients not having their voicemail set up. You're playing phone tag. They say, ‘Just send me a text,’ and you can't. So I think that is what started this [idea for text messaging].” *–Medical provider* |
